# Genome-Wide Analysis for Early Growth-Related Traits of the Locally Adapted Egyptian Barki Sheep

**DOI:** 10.3390/genes12081243

**Published:** 2021-08-13

**Authors:** Ibrahim Abousoliman, Henry Reyer, Michael Oster, Eduard Murani, Ismail Mohamed, Klaus Wimmers

**Affiliations:** 1 Leibniz Institute for Farm Animal Biology, Wilhelm-Stahl-Allee 2, 18196 Dummerstorf, Germany; abou-soliman@fbn-dummerstorf.de (I.A.); reyer@fbn-dummerstorf.de (H.R.); oster@fbn-dummerstorf.de (M.O.); murani@fbn-dummerstorf.de (E.M.); 2 Desert Research Center, Department of Animal and Poultry Breeding, 1 Mathaf El-Matareya St., El-Matareya, Cairo 11753, Egypt; ssmm_ismail@yahoo.com; 3 Faculty of Agricultural and Environmental Sciences, University of Rostock, Justus-von-Liebig-Weg 7, 18059 Rostock, Germany

**Keywords:** Barki sheep, growth, birth weight, weaning weight, indigenous sheep, lamb, SNP chip

## Abstract

Sheep play a critical role in the agricultural and livestock sector in Egypt. For sheep meat production, growth traits such as birth and weaning weights are very important and determine the supply and income of local farmers. The Barki sheep originates from the northeastern coastal zone of Africa, and due to its good adaptation to the harsh environmental conditions, it contributes significantly to the meat production in these semi-arid regions. This study aimed to use a genome-wide SNP panel to identify genomic regions that are diversified between groups of individuals of Egyptian Barki sheep with high and low growth performance traits. In this context, from a phenotyped population of 140 lambs of Barki sheep, 69 lambs were considered for a genome-wide scan with the Illumina OvineSNP50 V2 BeadChip. The selected lambs were grouped into divergent subsets with significantly different performance for birth weight and weaning weight. After quality control, 63 animals and 40,383 SNPs were used for analysis. The fixation index (*F_ST_*) for each SNP was calculated between the groups. The results verified genomic regions harboring some previously proposed candidate genes for traits related to body growth, i.e., *EYA2*, *GDF2*, *GDF10*, *MEF2B*, *SLC16A7*, *TBX15*, *TFAP2B*, and *TNNC2*. Moreover, novel candidate genes were proposed with known functional implications on growth processes such as *CPXM2* and *LRIG3*. Subsequent association analysis showed significant effects of the considered SNPs on birth and weaning weights. Results highlight the genetic diversity associated with performance traits and thus the potential to improve growth traits in the Barki sheep breed.

## 1. Introduction

Breeding is one of the main drivers affecting allele frequencies, leading to genomic regions with genetic differentiation, thus contributing to the genetic diversity of livestock species [1]. Many studies analyzing the genetic architecture of complex traits using genome-wide SNP data have been conducted in different livestock species in the last decade. In ruminants, these studies have detected candidate genes related to growth, muscle conformation, adaptation, and reproduction traits in sheep [2,3,4,5,6]; milk production, reproduction, body constitution, muscle development, coat color, and thermotolerance in cattle [7,8]; and adaptation, coat color, milk composition, and growth traits in goats [9,10]. To identify the corresponding genomic regions, several methods are available that are capable of analyzing genetic variation within a population and between populations or groups of individuals. The calculation of fixation index (*F_ST_*) of Weir and Cockerham (1984) is one of the most popular methods to analyze population structure in this framework. The *F_ST_* approach measures genomic differentiation between two or more populations, breeds, or divergent lines and subgroups in the same breed, depending on allele frequency, and is thus able to identify highly differentiated alleles that undergo selection, while being unbiased for sample size [7,11].

Growth traits are very important in sheep breeding and considerably affect the resource efficiency and breeder’s profit. Growth traits, like other quantitative traits, are controlled by the complex genetic background of the animal as well as environmental factors, such as feed and herd management. Body weight gain has a moderate to high heritability [12,13,14] and is one of the main indices of selection, especially for meat type breeds. However, it also influences wool production and the reproduction performance of sheep [15]. Body weight gain can be monitored at birth or at other animal life stages and largely determines the amount of income from sheep meat production. Weight measurement at birth represents the earliest indicator of growth performance and related traits [16]. Various genetic but also non-genetic factors affect the birth weight, such as the dam’s weight, age, and nutrient supply during pregnancy [17]. The heritability of birth weight was estimated to be 0.39 and 0.31 in Iranian Mehraban and Lori–Bakhtiari sheep breeds, respectively. For weaning weight, heritability was estimated to be 0.25 for weaning weight in Iranian Mehraban sheep [17]. Heritabilities for birth weight and weaning weight were 0.19 and 0.20 in Barki sheep, respectively [18]. For Barki sheep-breeding in particular, high weight at birth is not a primary goal; more importance is given to achieving higher weaning weights [18]. This is also related to the fact that problems for the dam or the newborn lamb should be avoided, such as dystocia during the birth process. In fact, Barki sheep show large differences in growth traits, ranging from 2.4 to 5.0 kg for birth weight with an average of 3.7 kg and from 5.2 to 28.8 kg for weaning weight with an average of 13.8 kg [19]. In general, although Barki sheep are highly adapted to the harsh environmental conditions of northeast Africa and have a high value for farmers in this region, it remains difficult to obtain a large number of genotyped and comprehensively phenotyped animals due to the smallholder sheep farming systems in Egypt [20]. Improving growth traits in sheep using genetics is promising and is being implemented in different sheep populations but would further benefit from an understanding of the underlying biological processes and functions. The aim of the current study was to gain insight into diversified genomic regions for growth traits in the Barki sheep breed and to derive potential candidate genes that are functionally related to the traits of interest.

## 2. Materials and Methods

### 2.1. Animals and Phenotypes

In this study, a population of 140 lambs (55 males and 85 females) from single births of the Egyptian Barki sheep was considered for investigation of the genomic regions and candidate genes for growth traits comprising birth weight and weaning weight. Animals were raised in the farms of the Desert Research Centre (DRC), Ministry of Agriculture, Egypt. They were kept under an intensive breeding system in semi-open yards. The lambs were from one breeding season and were offspring of 10 rams. Birth weight within 12 h after parturition and weaning weight after a lactation period of 3 months (90 days) were recorded for every lamb using an electronic scale. From birth to weaning age, the lambs were suckled only on their mother’s milk daily. Fresh water was offered two times daily to lambs ad libitum. The experiment was conducted in accordance with all ethics and animal rights (DRC), considering all regulations in conformity with the European Union Directive for the protection of experimental animals (2010/63/EU).

### 2.2. Genotyping and Quality Control

For each lamb, blood was sampled from the jugular vein in EDTA-containing tubes and stored at −80 °C until DNA extraction. DNA extraction was performed according to the manufacturer’s instructions with the G-spin Total DNA Extraction kit (iNtRON Biotechnology, Seoul, Korea). Out of the entire population of 140 animals, 69 lambs with considerable differences in the respective growth trait were genotyped using the Illumina OvineSNP50 V2 BeadChip (Illumina, San Diego, CA, USA). The relative identity-by-descent (IBD) was calculated for all pairs of lambs. The genetic relatedness average was 0.15. The iScan Reader (Illumina) was used to image the raw signal intensities of the 53,516 SNPs on the chip, which were subsequently converted into genotypes with the GenomeStudio software (version 2.0). The samples with call rates < 90% were removed from further analysis. The SNPs with genotype call rates < 98%, minor allele frequencies (MAF) < 0.05, in high linkage disequilibrium (r^2^ > 0.5) within windows of 50 SNPs, and significant deviation from Hardy–Weinberg equilibrium at *p* < 10^−6^ were removed from the analysis. JMP Genomics software (version 9) was used for quality control. A total of 63 animals and 40,383 SNPs remained and passed the quality control. Base pair positions and names of SNP markers were updated to the latest version of the ovine genome (Oar_v3.1 accessed on 6 July 2020). SNPs not located on autosomes and lacking reference SNP (rs) identifiers were excluded. Based on the SNP data, the genomic relationship matrix of lambs was calculated using SNPRelate R package (version 1.24.0) and visualized in a principal component plot (Supplementary Materials Figure S1).

### 2.3. F_ST_ Analysis and Screening for Candidate Genes

**Figure 1:**
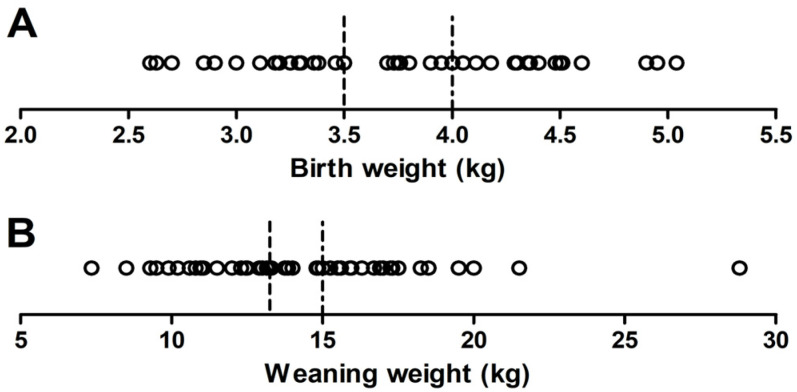
Distribution of lambs according to their birth weight (**A**) and weaning weight (**B**). The dashed lines indicate the thresholds for classification of animals into low and high groups.

For each of the traits, birth weight and weaning weight, the genotyped animals were divided into two subgroups, each representing the extreme phenotypes for the respective trait with significant differences between the groups (Figure 1). The Student’s *t*-test was used to compute the differences between the means of high and low groups. The fixation index (*F_ST_*) for each SNP was calculated between the groups low birth weight (LBW)—high birth weight (HBW) and low weaning weight (LWW)—high weaning weight (HWW) by the SNPRelate R package (version 1.24.0) using the Weir and Cockerham method [21,22]. *F_ST_* values were Z-transformed using the following equation Z(*F_ST_*) = (*F_ST_* − μ *F_ST_*)/ϭ *F_ST,_* where μ *F_ST_* is the overall mean of *F_ST_* values and ϭ *F_ST_* is the standard deviation of *F_ST_* values. In addition, an SNP-specific baseline *F_ST_* estimate was calculated to evaluate the results of the *F_ST_* analysis. This estimation was based on a random permutation of the group assignment with 10,000 iterations. Subsequently, the distribution of SNP-specific *F_ST_* values was used to calculate permutation-based *p*-values for the intended group comparisons (LBW–HBW and LWW–HWW) using the statmod R package [23]. Using the genomic inflation factor lambda (λBW = 1.19, λWW = 1.72), genomic control corrections were made and permutation *p*-values were adjusted. Only the SNPs having the top 0.05% of Z(*F_ST_*) values were selected for further analysis. Manhattan plots of Z(*F_ST_*) value for each SNP were constructed using qqman package (version 0.1.4) in R software. To identify the candidate genes, the Ensembl database was used to select the genes within 2 Mb windows around high Z(*F_ST_*) SNPs [24]. Genes harboring a highlighted Z(*F_ST_*) SNP were considered as positional candidate genes. Functional candidate genes in the respective genomic region were identified according to their functional relation with the phenotypes, employing available gene annotations from the GeneCards (http://www.genecards.org; accessed on 19 May 2021) and Uniprot (http://www.uniprot.org; accessed on 19 May 2021) databases. Kyoto Encyclopedia of Genes and Genomes (KEGG, https://www.kegg.jp/; accessed on 23 July 2021) pathway enrichment analysis of the functional, positional, and closest up- and downstream-located genes based on SNPs that exceeded the threshold was performed using ClueGO plugin with the Cytoscape software (version 3.6.1) [25,26].

### 2.4. Marker–Trait Association Analysis

The association analysis was carried out to test the effect of the genotypes of the selected SNPs on the phenotypes of birth and weaning weight by JMP Genomics software (version 9) using the QK mixed model procedure considering the genomic relationship matrix. In addition, the statistical model included the sex of lamb as fixed effect (two levels). *p* ≤ 0.05 was considered significant, *p* ≤ 0.01 highly significant, and *p* > 0.05 not significant.

## 3. Results

### 3.1. Phenotypic Data of Growth Traits

Descriptive statistics of birth weight and weaning weight of Barki lambs with divergent performance in the respective trait are shown in Table 1.

### 3.2. Detection of Genomic Regions and Candidate Genes

**Figure 2:**
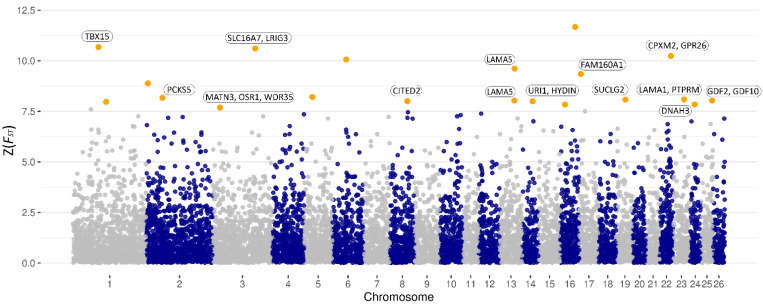
Manhattan plot of the Z(*F_ST_*) values for each single nucleotide polymorphism (SNP) between Barki sheep groups divergent in birth weight (LBW–HBW). Orange dots represent SNPs that passed the cutoff threshold at 99.95% of the percentile distribution (Z(*F_ST_*) ≥ 7.68).

For the detection of genomic regions and candidate genes of growth traits, subgroups with distinct differences in the respective traits were analyzed. In particular, Z(*F_ST_*) values were calculated to explore genomic differences between groups using genome-wide distributed SNPs. Figure 2 shows a Manhattan plot of SNP-specific Z(*F_ST_*) values for birth weight and highlighted genomic regions and SNPs with the highest Z(*F_ST_*) values. Obtained SNPs and regions were distributed on chromosomes 1, 2, 3, 5, 6, 8, 13, 14, 16, 17, 19, 22, 23, 24, and 25 (cutoff Z(*F_ST_*) ≥ 7.68, Table 2). Genomic regions highlighted by the selected SNPs were mined for positional and functional candidate genes (Table 2). This analysis yielded eight positional candidate genes comprising *PCKS5*, *WDR35*, *LAMA5*, *HYDIN*, *FAM160A1*, *GPR26*, *PTPRM*, and *DNAH3*. Moreover, a total of 13 functional candidate genes in the indicated genomic regions were identified, which are known to affect birth weight or are involved in the development process during the embryonic stage and after birth. These comprise *TBX15, SLC16A7, LRIG3, MATN3, OSR1, CITED2, LAMA5, URI1, SUCLG2, CPXM2, LAMA1, GDF2*, and *GDF10*. Among the vital processes to which these genes contribute is the development of body organs, such as limbs, liver, lungs, brain, skeletal system, and muscles. Except for the SNPs indicating the genomic regions harboring *BRINP1*, *CITED2*, *CPXM2*, *GDF2*, and *GDF10*, the marker–trait association analysis confirmed a significant relationship of the candidate loci with birth weight in the Barki lamb population (Table 2, Supplementary Materials Table S1).

**Figure 3:**
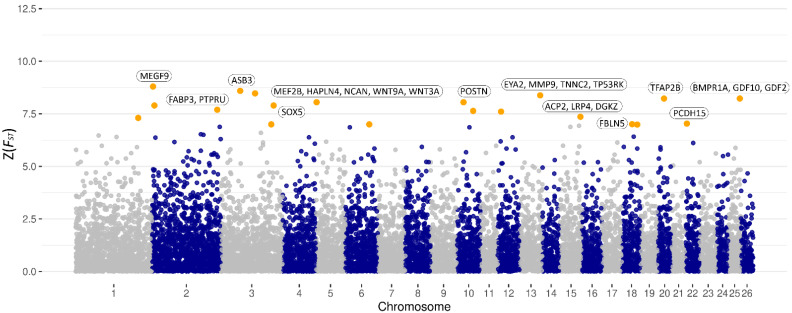
Manhattan plot of the Z(*F_ST_*) values for each single nucleotide polymorphism (SNP) of weaning weight. Orange dots represent SNPs that passed the cutoff threshold at 99.95% of the percentile distribution (Z(*F_ST_*) ≥ 6.99).

For weaning weight, 20 SNPs, which reached the threshold Z(*F_ST_*) value at 99.95% of the percentile distribution, were identified (cutoff Z(*F_ST_*) ≥ 6.99, Table 3). These SNPs indicated 13 genomic regions distributed on chromosomes 1, 2, 3, 5, 6, 10, 12, 13, 15, 18, 20, 22, and 25, as illustrated in Figure 3. Considering these regions, seven positional candidate genes, directly tagged by one of the identified SNPs, were detected. These genes are *ASB3, SOX5, TP53RK, DGKZ, FBLN5, PCDH15,* and *GLUD1*. Moreover, 19 functional candidate genes were identified in the respective genomic regions for weaning weight including *MEGF9, PTPRU, FABP3, MEF2B, HAPLN4, NCAN, WNT9A, WNT3A, POSTN, EYA2, MMP9, TNNC2, ACP2, LRP4, FBLN5, TFAP2B, BMPR1A, GDF2,* and *GDF10*. Considering the SNPs indicative for these candidate genes, most of the SNPs appeared to be significantly associated with weaning weight (Table 3, Supplementary Materials Table S2). Considering all SNPs that exceeded the threshold for birth and weaning weights, results of the enrichment analysis of the functional, positional, and closest up- and downstream located genes within 2 Mb are presented (Supplementary Materials Figure S2).

## 4. Discussion

In this study, we used the Ovine SNP50 V2 BeadChip to identify the genomic differences between groups of Barki sheep that differed significantly in growth traits (birth and weaning weight). The average birth weight of Barki lambs was similar to those of lambs of the native Egyptian breeds, such as Rahmani and Ossimi breeds, with 3.73 and 3.9 kg. However, in terms of weaning weight, Rahmani and Ossimi lambs are slightly heavier than Barki lambs with average weights of 17.63 and 14.05 kg, respectively [27,28]. Moreover, birth weight (5.08 kg) and weaning weight (29.8 kg) of Romney lambs, as one of the worldwide economically important meat-type breeds, were reported to be higher than the weights of Barki sheep [29]. In order to elucidate genetic contributions, genetic differentiations between the subgroups were investigated by the calculation of SNP-specific Z(*F_ST_*) values. Considering birth weight and weaning weight, identified genomic regions on chromosomes 2, 3, 5, 15, 16, 18, 22, 24, and 25 were previously proposed in the sheep QTL database to be associated with some growth traits such as body weight at different ages of lambs and average daily gain [30]. A total of 15 genomic regions were found to exhibit divergent allele frequencies for birth weight in Barki lambs. Regions on chromosomes 1, 3, and 16 are proposed to be the most promising regions, since they enclose four SNPs with the highest Z(*F_ST_*) values. Within the genomic region on chromosome 1 at 95.3 Mb, T-box 15 (*Tbx15*) appears to be the most promising functional candidate gene. *Tbx15* gene is a member of T-box gene family, which encodes for transcription factors. T-box genes play a critical role in the development of different tissues and organs in vertebrates and invertebrates, such as the skeletal system during the embryonic stage [31]. The expression of Tbx15 was reported in the development of forelimb and hind limb buds in chick and mouse [32,33]. Within chromosome 3, the solute carrier family 16 member 7 (*SLC16A7*) gene at 159.6 Mb and the leucine-rich repeats and immunoglobulin-like domains protein 3 (*LRIG3*) gene at 160.4 Mb were proposed as additional promising candidates for birth weight. *SLC16A7* was reported to have a role in skeletal muscle as a lactate cotransporter [34]. *LRIG3* is known to be involved in placenta and embryonic development in pigs [35]. Moreover, the carboxypeptidase-like protein X2 encoding gene (*CPXM2*) located on chromosome 22 at 42.4 Mb plays an important role in growth processes. It was reported to be one of the genes that regulate backfat thickness at different life stages in pigs [36].

Within the genomic region on chromosome 5 at 3.1 Mb, the myocyte enhancer factor-2B (*MEF2B*) gene was proposed as a functional gene for weaning weight. This gene encodes a member of the myocyte enhancer factor-2 family (*MEF2A, MEF2B, MEF2C,* and *MEF2D*), which plays an important and critical role in cell development, embryonic development, muscle tissue growth, and development processes [37,38]. Polymorphism in the 3′-UTR of the *MEF2B* gene showed a significant correlation with some growth traits in New Ujumqin Sheep such as body weight and chest girth at 4 and 6 months [39]. Previous studies revealed a significant association between *MEF2B* and skeletal muscle satellite cell and reproductive traits in pigs [40] as well as diameter of muscle fibers in goats [41]. The SNP indicating the *MEF2B* region was found to be significantly associated with weaning weight. Within chromosome 13, the eyes absent homolog 2 (*EYA2*) and the fast skeletal muscle troponin C (*TNNC2*) genes were revealed as candidates for weaning weight. In Ethiopian sheep, EYA2 was proposed as a candidate for embryonic development of tendons, bones, and cartilages [42]. *TNNC2* plays a critical role in skeletal muscle contraction, modulates the Ca2+-activation characteristics of muscle fibers [43], and is highly expressed during the myoblast differentiation and skeletal muscle development [44]. Previous studies on *TNNC2* reported a significant association with growth traits in porcine skeletal muscles [45] and with carcass weight and marbling score in three native sheep breeds [46].

Within the region on chromosome 20 at 23.4 Mb, the transcription factor AP-2B (*TFAP2B*) gene was proposed as a functional candidate for weaning weight. *TFAP2B* was documented in vertebrates and invertebrates and has a critical role during embryonic development [47]. The function of *TFAP2B* in the development of craniofacial structures, limb formation, and kidney and skin development was reported in mice [48].

Based on the indicated genomic regions of birth weight and weaning weight, some functional candidate genes were proposed such as growth differentiation factors 2 and 10 genes (*GDF2, GDF10*) on chromosome 25. *GDF2* and *GDF10* are members of the transforming growth factor-β (TGF- β) superfamily and the bone morphogenetic protein family (BMP), also known as bone morphogenetic protein 9 and 3B (*BMP9, BMP3B*) [49]. Previous studies reported a significant correlation between *GDF2* and *GDF10* genes and weaning weight in New Zealand dual-purpose sheep [50]. *GDF10* regulates cell growth and differentiation in embryonic and adult tissues. Genetic polymorphism in the bovine *GDF10* gene showed a significant effect on some body measurements in Chinese indigenous cattle [51]. Results of the marker–trait association analysis confirmed the significant effect of the majority of selected SNPs on both birth weight and weaning weight and reflected the contribution of these SNPs to the phenotypic differences between high and low groups of these traits. Results of the enrichment analysis revealed the contribution of the selected genes in some physiological pathways related to growth performance such as animal organ formation, regulation of embryonic development, and skeletal muscle development, which confirm the critical role of these genes in growth processes. However, the robustness of association analysis is a matter of sample size [52]; the results of this study require validation in an independent population and with a larger sample size.

## 5. Conclusions

The genome-wide SNPs analysis revealed a number of genomic regions containing putative QTL for birth weight and weaning weight. The QTL regions cover a number of promising functional candidate genes such as *CPXM2, EYA2, GDF2, GDF10, LRIG3, MEF2B, SLC16A7, TBX15, TFAP2B,* and *TNNC2*, which deserve further investigation, due to their relation to biological processes, including metabolism, body growth, organ morphogenesis, skeletal muscle development, and cell proliferation and differentiation. Moreover, the marker–trait association analysis revealed a significant relationship of the considered SNPs to the studied traits. Our findings provide valuable information for a better understanding of the genetics of early growth-related traits and might contribute to the improvement of these traits in the Barki sheep breed.

## Figures and Tables

**Table 1:** Descriptive statistics of lamb growth traits.

Trait	Abbreviation	Group *	N	Mean	SD	Min	Max	*p*-Value **
Birth weight (kg)	BW	HBW	31	4.3	0.3	4.0	5.0	*p* < 0.001
		LBW	24	3.2	0.3	2.6	3.5	
Weaning weight (kg)	WW	HWW	28	18.1	2.5	15.0	28.8	*p* < 0.001
		LWW	23	11.2	1.7	7.4	13.3	

* HBW = High birth weight, LBW = Low birth weight, HWW = High weaning weight, LWW = Low weaning weight. ** = *p*-value computed using *t*-test, SD = Standard deviation.

**Table 2:** Genomic positions of SNPs that exceeded the cutoff threshold (Z(*F*_ST_) ≥ 7.68) for divergence in birth weight and adjacent candidate genes within a 2 Mb window.

Rs Identifier	Chr	Position	MAF	Z(*F*_ST_)	Permutation *p* Value ^1^	Association *p* Value ^2^	Candidate Genes ^3^
rs426652102	1	95369397	0.335	10.68	0.0006	0.004	***TBX15***, *SPAG17*
rs409727057	1	124117118	0.220	7.97	0.001	0.003	*CLDN17*, *GRIK1*
rs423222301	2	5472326	0.301	8.88	0.0006	0.015	*BRINP1*, *TLR4*
rs419514091	2	60044110	0.369	8.17	0.0006	0.116	*PCKS5*, *RFK*, *ENSOARG00000012533*
rs428614465	3	27732721	0.492	7.68	0.0009	<0.0001	***MATN3***, ***OSR1***, *WDR35*, *TTC32*
rs417060060	3	160252310	0.446	10.61	0.0003	0.001	*SLC16A7*, *LRIG3*
rs413547561	5	31477570	0.481	8.21	0.001	0.144	*PRR16*, *FAM170A*
rs413364871	6	51650705	0.301	10.06	0.0003	0.006	*--*
rs427385309	8	64075259	0.276	8.00	0.0006	0.156	***CITED2***, *U5*, *NMBR*
rs406649973	13	54940415	0.444	9.61	0.0003	0.041	***LAMA5***, *CDH28*, *CDH4*
rs419112095	13	54219758	0.295	8.03	0.0009	0.166	***LAMA5***, *RPS21*, *ADRM1*
rs410323459	14	39415649	0.328	8.00	0.001	0.0006	***URI1***, *HYDIN*, *CMTR2*, *POP4*
rs423237115	16	56144068	0.412	11.67	0.0003	0.014	*MYO10*
rs413049228	16	18186560	0.134	7.84	0.009	0.058	*ZSWIM6*, *SMIM15*
rs413966946	17	6026379	0.465	9.34	0.0006	0.006	*FAM160A1*, *GATB*, *SH3D19*
rs430430903	19	33088813	0.289	8.08	0.0006	0.046	***SUCLG2***, *ENSOARG00000010424*
rs417719085	22	42323039	0.444	10.23	0.0003	0.002	***CPXM2***, *GPR26*, *ACADSB*
rs412781362	23	41177245	0.127	8.07	0.0009	0.001	***LAMA1***, *PTPRM*, *RAB12*
rs421209784	24	18748352	0.086	7.84	0.0009	0.001	*DNAH3*, *LYRM1*, *TMEM159*
rs429736586	25	41911462	0.348	8.03	0.0003	0.064	***GDF2***, ***GDF10***, *PTPN20*

^1^ *p*-value resulting from permutation-based test of *F_ST_* values and subsequent adjustment for genomic inflation; ^2^ *p*-value resulting from association analysis of the respective SNP with birth weight; ^3^ Gene names in **bold** = functional candidate genes, underlined = positional candidate genes, only *italic* = closest up- and downstream-located genes within a 2 Mb window, Chr = chromosome, MAF = minor allele frequency, Z(*F_ST_*) = SNP-specific Z-transformed fixation index.

**Table 3:** Genomic positions of SNPs that exceeded the cutoff threshold (Z(*F*_ST_) ≥ 6.99) for divergence in weaning weight and adjacent candidate genes within a 2 Mb window.

Rs Name	Chr	Position	MAF	Z(*F*_ST_)	Permutation *p*-Value ^1^	Association *p*-Value ^2^	Candidate Genes ^3^
rs401497638	1	226047403	0.274	7.30	0.170	0.002	*IL12A*, *IQCJ*
rs402362274	2	4014386	0.194	8.80	0.003	0.015	***MEGF9***, *CDK5RAP2*, *BRINP1*
rs427650461	2	8985061	0.259	7.90	0.003	0.018	*TNFSF15*, *TMEM268*
rs405054059	2	236085906	0.354	7.69	0.004	0.177	***FABP3***, *MATN1*, ***PTPRU***
rs420573745	3	70227376	0.500	8.59	0.003	0.077	*ASB3*, *CHAC2*
rs422502823	3	124010853	0.408	8.47	0.003	0.039	*MGAT4C*, *C12orf50*
rs425747978	3	191139722	0.269	7.90	0.003	0.067	*SOX5*, *BCAT1*, *ETNK1*
rs410754805	3	182669294	0.341	7.00	0.003	0.044	*RESF1*, *AMN1*
rs429678680	5	3076397	0.224	8.10	0.003	0.278	***MEF2B***, ***HAPLN4***, ***NCAN***, ***WNT9A***, ***WNT3A***, *PRSS38*, *SNAP47*
rs418926568	6	86505675	0.381	7.00	0.004	0.120	*SLC4A4*, *GC*
rs401888979	10	25115607	0.219	8.10	0.003	0.002	***POSTN***, *RFXAP*, *SERTM1*
rs428497629	10	59974520	0.263	7.64	0.004	0.006	*SLITRK1*, *SLITRK6*
rs426943634	12	12266633	0.436	7.60	0.003	0.009	*RGS18*, *BRINP3*
rs413169429	13	74924747	0.303	8.38	0.003	0.009	***EYA2***, ***MMP9***, ***TNNC2***, *TP53RK*, *SLC13A3*, *SLC2A10*
rs411451096	15	74597812	0.399	7.36	0.003	0.128	***ACP2***, ***LRP4***, *DGKZ*, *CREB3L1*, *MDK*
rs430684800	18	36936501	0.322	7.01	0.003	0.009	*NOVA1*, *FOXG1*
rs426036565	18	55537048	0.414	6.99	0.003	0.495	***FBLN5***, *TC2N*, *TRIP11*
rs410079568	20	23394498	0.470	8.23	0.003	0.143	***TFAP2B***, *PKHD1*
rs421690996	22	5090223	0.345	7.03	0.003	0.026	*PCDH15*, *U3*, *SYCE1*
rs400432841	25	41241866	0.459	8.23	0.003	0.039	***BMPR1A***, ***GDF10***, ***GDF2***, *GLUD1*, *U6*, *SHLD2*

^1^ *p*-value resulting from permutation-based test of *F_ST_* values and subsequent adjustment for genomic inflation; **^2^** *p*-value resulting from association analysis of the respective SNP with weaning weight; ^3^ Gene names in **bold** = functional candidate genes, underlined = positional candidate genes, only *italics* = closest up- and downstream-located genes within a 2 Mb window, Chr = chromosome, MAF = minor allele frequency, Z(*F*_ST_) = SNP-specific Z-transformed fixation index.

## Data Availability

Genotypic and phenotypic information has been deposited at Open Science Framework (https://osf.io/qj29r/; accessed on 19 November 2020).

## Supplementary Materials

The following tables are available online at www.mdpi.com/article/10.3390/genes12081243/s1. Table S1: Association of selected SNP genotypes with birth weight in Egyptian Barki lambs (Mean ± SE); Table S2: Association of selected SNP genotypes with weaning weight in Egyptian Barki lambs (Mean ± SE); Figure S1: Principal component plot of the genomic relationship of animals displaying the classification into high and low groups for birth weight (frame color) and weaning weight (fill color); Figure S2: KEGG pathway enrichment analysis of the functional, positional, and closest up- and downstream located genes within a 2 Mb window.
