# Supplementary figures and images for: Genome-Wide Analysis for Early Growth-Related Traits of the Locally Adapted Egyptian Barki Sheep
Source: Genes (Basel). 2021 Aug 13;12(8):1243. doi: 10.3390/genes12081243 (PMC8394750; doi:10.3390/genes12081243)

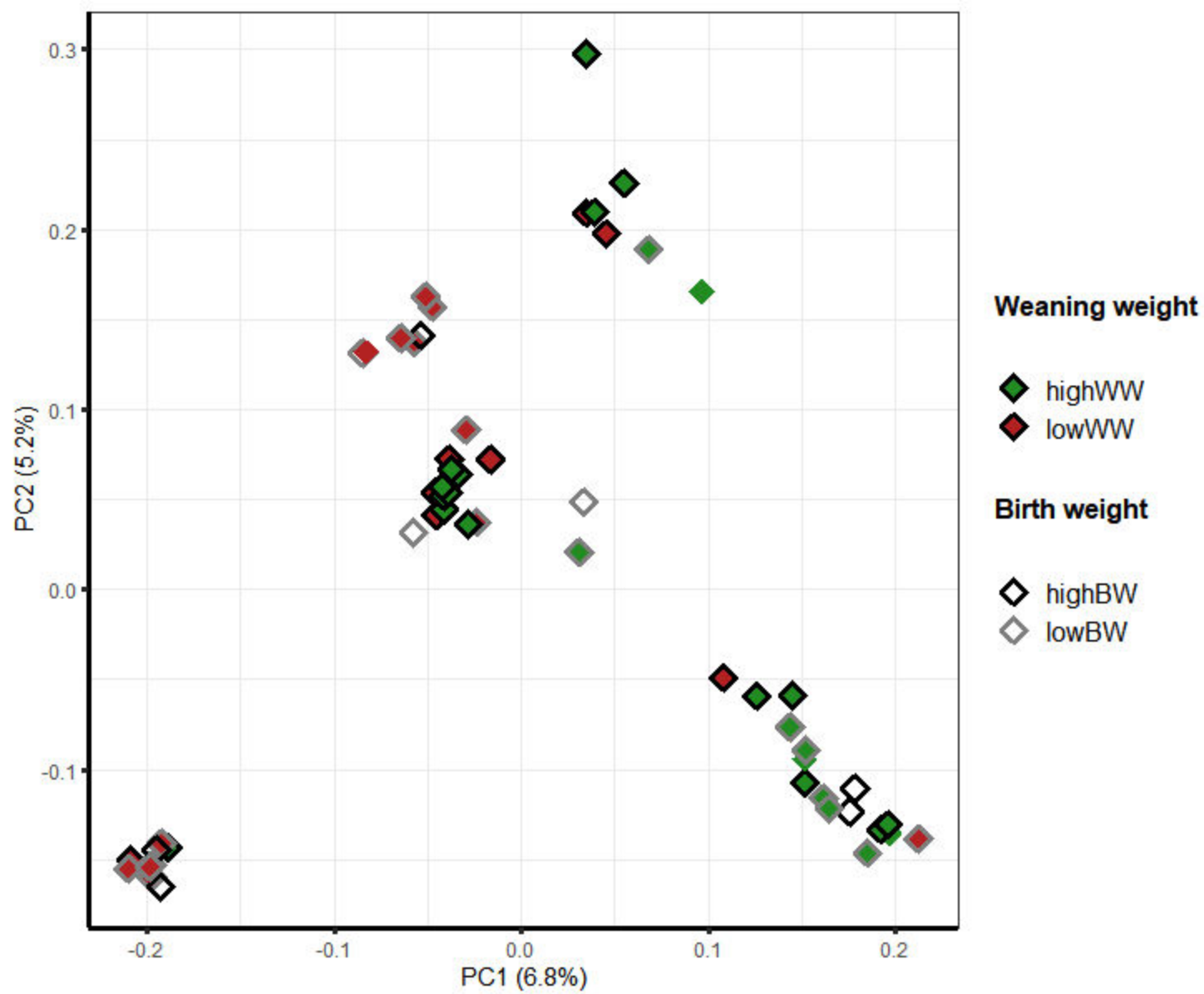

Supplement: Supplementary file 1 [file genes-12-01243-s001.zip › Supplemntary Figure S1.pdf]

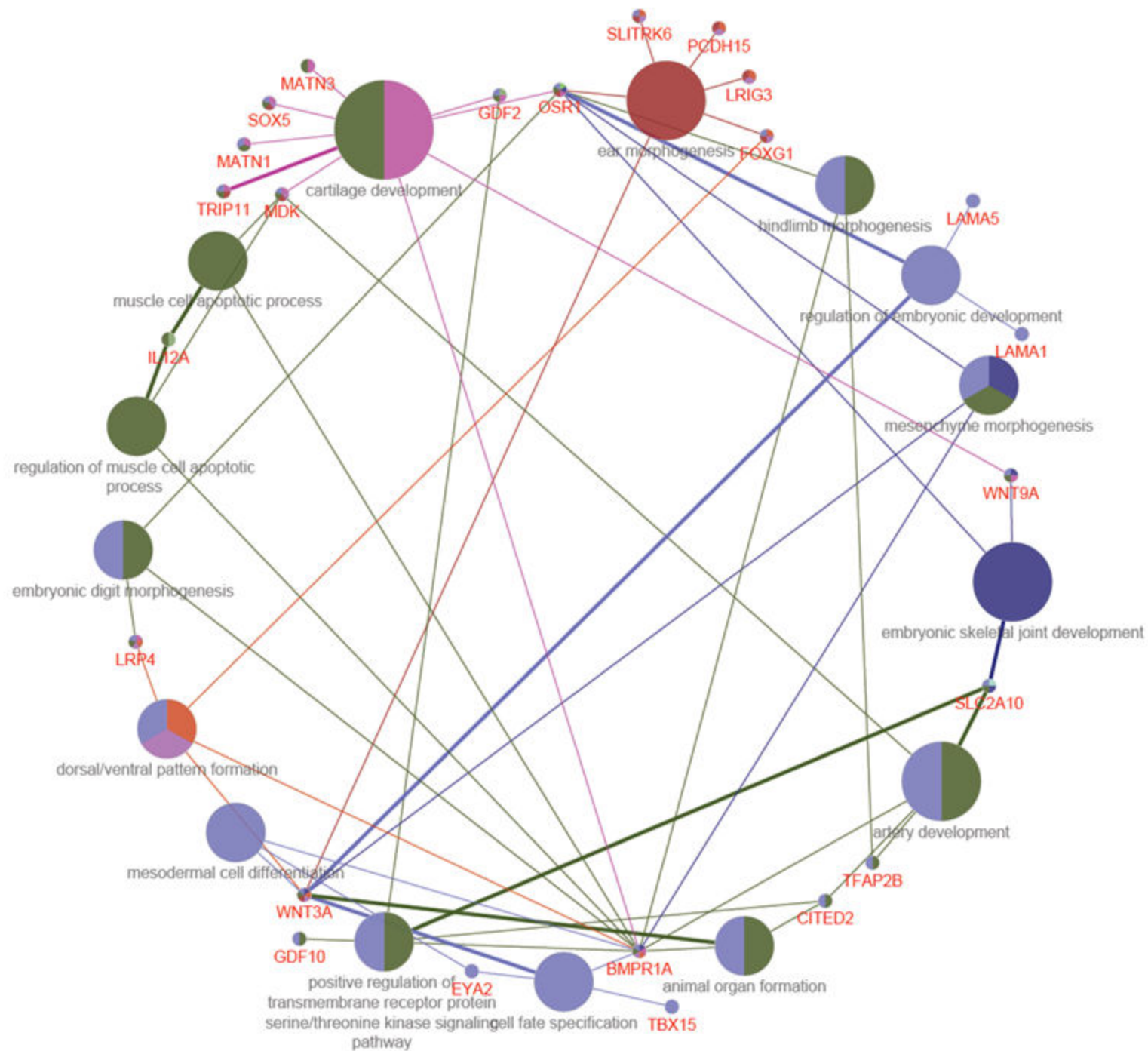

Supplement: Supplementary file 1 [file genes-12-01243-s001.zip › Supplemntary Figure S2.pdf]
